# A Proteolytic Cascade Controls Lysosome Rupture and Necrotic Cell Death Mediated by Lysosome-Destabilizing Adjuvants

**DOI:** 10.1371/journal.pone.0095032

**Published:** 2014-06-03

**Authors:** Jürgen Brojatsch, Heriberto Lima, Alak K. Kar, Lee S. Jacobson, Stefan M. Muehlbauer, Kartik Chandran, Felipe Diaz-Griffero

**Affiliations:** Albert Einstein College of Medicine, Department of Microbiology and Immunology, Bronx, New York, United States of America; Karolinska Institutet, Sweden

## Abstract

Recent studies have linked necrotic cell death and proteolysis of inflammatory proteins to the adaptive immune response mediated by the lysosome-destabilizing adjuvants, alum and Leu-Leu-OMe (LLOMe). However, the mechanism by which lysosome-destabilizing agents trigger necrosis and proteolysis of inflammatory proteins is poorly understood. The proteasome is a cellular complex that has been shown to regulate both necrotic cell death and proteolysis of inflammatory proteins. We found that the peptide aldehyde proteasome inhibitors, MG115 and MG132, block lysosome rupture, degradation of inflammatory proteins and necrotic cell death mediated by the lysosome-destabilizing peptide LLOMe. However, non-aldehyde proteasome inhibitors failed to prevent LLOMe-induced cell death suggesting that aldehyde proteasome inhibitors triggered a pleotropic effect. We have previously shown that cathepsin C controls lysosome rupture, necrotic cell death and the adaptive immune response mediated by LLOMe. Using recombinant cathepsin C, we found that aldehyde proteasome inhibitors directly block cathepsin C, which presumably prevents LLOMe toxicity. The cathepsin B inhibitor CA-074-Me also blocks lysosome rupture and necrotic cell death mediated by a wide range of necrosis inducers, including LLOMe. Using cathepsin-deficient cells and recombinant cathepsins, we demonstrate that the cathepsins B and C are not required for the CA-074-Me block of necrotic cell death. Taken together, our findings demonstrate that lysosome-destabilizing adjuvants trigger an early proteolytic cascade, involving cathepsin C and a CA-074-Me-dependent protease. Identification of these early events leading to lysosome rupture will be crucial in our understanding of processes controlling necrotic cell death and immune responses mediated by lysosome-destabilizing adjuvants.

## Introduction

While research on programmed cell death has focused mainly on apoptosis, recent studies have highlighted the relevance of necrotic cell death in many biological and immunological processes. For example, necrotic cell death has been implicated in microbial pathogenesis, septic shock and adaptive immune responses [1], [2], [3], [4], [5], [6], [7], [8]. While apoptotic cells retain their intracellular content, necrotic cell death is characterized by plasma membrane impairment and the release of intracellular factors driving inflammatory responses. Specifically, the necrotic release of uric acid, MHGB1, double-stranded DNA, and ATP has been linked to immune responses mediated by necrotic cell death inducers [9], [10], [11], [12], [13], [14].

While necrosis was originally considered a traumatic disregulated process caused by direct chemical or radiologic insult [15], recent studies indicate that necrotic cell death is, like apoptosis, a highly regulated process with inducer-specific checkpoints [7], [16]. For example, pyroptosis, the best-characterized form of necrosis, requires caspase-1 activation and inflammasome signaling [16], [17], [18], [19], [20], [21]. The second form of necrotic cell death, necroptosis, is induced by specific death receptors, such as TNF-α and Trail, in the presence of caspase inhibitors [22],[23]. Recent studies indicate that lysosome-destabilizing agents mediate a third form of programmed necrosis, termed as lysosome-mediated necrosis (LMN) [9], [24], [25], [26], [27]. Inducers of LMN include alum, silica crystals, cholesterol crystals, amyloid proteins, and the dipeptide methyl ester Leu-Leu-OMe (LLOMe) [9], [24], [28].

Though all forms of necrotic cell death have been linked to inflammation, only LMN has specifically been linked to the induction of the adaptive immunity [9], [11],[28]. LMN is characterized by early lysosome-rupture followed by plasma membrane impairment and proteolysis of low-molecular-weight point proteins [11], [24]. As a result, several key inflammatory proteins, including caspase-1, IL-1β and IL-18, are degraded, decreasing their signal [11], [24]. Prior studies have elicited three cathepsins as critical regulators of lysosome-mediated necrosis: cathepsin C is crucial for LLOMe-mediated necrosis, while cathepsins B and S are necessary for alum-mediated necrosis [9], [11], [28]. The mechanism by which lysosome-destabilizing agents trigger proteolysis of cytosolic proteins and plasma membrane impairment remains unclear.

Previous studies have linked the proteasome system to proteolysis of inflammatory proteins and programmed cell death [29], [30], [31], [32]. The ubiquitin-proteasome pathway is a major proteolytic system in eukaryotic cells, and responsible for degrading proteins flagged by ubiquitin moieties [33]. The proteasome system is also a critical regulator of multiple forms of necrotic and apoptotic cell death [29], [30]. Specifically, the proteasome system controls lysosome rupture, necrotic cell death and proteolysis of inflammatory proteins mediated by the pyroptosis inducer anthrax lethal toxin (LT) [29], [31], [32]. Here we investigated the extent to which the proteasome also regulates cell death and proteolysis of cellular proteins in lysosome-mediated necrosis. Here we report that the aldehyde proteasome inhibitors, MG115 and MG132, block the degradation of proinflammatory proteins and necrotic cell death mediated by LLOMe. Using a combination of cellular, genetic, and biochemical models we present evidence lysosome-mediated necrosis is a proteasome-independent process, but that aldehyde proteasome inhibitors target cathepsin C, a protein previously shown to be critical for LLOMe-mediated lysosome rupture. In contrast to aldehyde proteasome inhibitors, the broad necrosis inhibitor CA-074-Me blocked LLOMe-induced lysosome rupture and necrotic ell death without targeting cathepsins C. Taken together our findings indicated that aldehyde proteasome inhibitors and CA-074-Me target distinct proteins involved in a proteolytic cascade preceding lysosome rupture and necrotic cell death mediated by lysosome-disrupting adjuvants.

## Results and Discussion

### The proteasome inhibitor MG115 inhibits cell death and proteolysis of inflammatory proteins mediated by the lysosome-destabilizing adjuvant LLOMe

**Figure 1:**
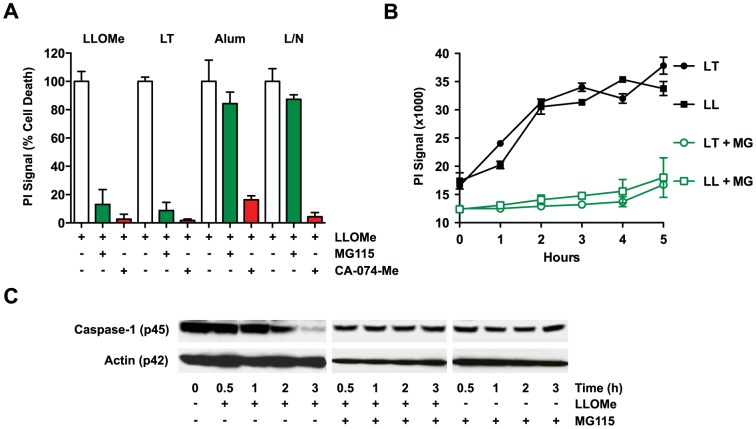
The proteasome inhibitor MG115 blocks LLOMe-mediated cell death and protein degradation. (A) BALB/c-derived macrophages were primed with 250 ng/ml LPS for 2 hours, and then treated with 2.5 mM LLOMe, anthrax lethal toxin (LT: 500 ng/ml PA and 250 ng/ml LF), alum (150 µg/ml), or 10 µM nigericin (LN) in the absence or presence of 100 µM MG115 or 100 µM CA-074-Me. Cell death was measured after 2 hours (except for alum, which was exposed for 8 hours) by propidium iodide exclusion. Relative levels are blotted with inducer alone adjusted to 100% cell death. (B) BALB/c-derived macrophages were exposed to 2.5 mM LLOMe (LL) or anthrax lethal toxin (500 ng/ml PA and 250 ng/ml LF) in the absence or presence of 100 µM MG115 (MG). Cell death was measured by propidium iodide exclusion. (C) BALB/c macrophages were exposed to 2.5 mM LLOMe in the absence or presence of 100 µM MG115. Cellular lysates were isolated at different time points after LLOMe exposure, and were subjected to immunoblotting and probed with anti-caspase-1 and actin antibodies.

Recent studies have demonstrated that necrotic cell death mediated by lysosome-destabilizing agents induces a strong adaptive immune response [9], [11], [28]. While necrotic cell death and proteolysis of inflammatory proteins have been linked to the immunogenicity of lysosome-disrupting adjuvants [9], [11], [12], [24], the underlying cellular mechanisms that control these processes are poorly understood. We have previously shown that the ubiquitin-proteasome system is critical for necrotic mediated by anthrax lethal toxin (LT), but not by other necrosis inducers, such as LPS/nigericin [29], [31], [32], [34]. To determine whether the proteasome also controls necrotic and proteolytic processes mediated by lysosome-destabilizing agents, we treated primary bone marrow-derived murine macrophages with the lysosome-destabilizing adjuvants alum and Leu-Leu-OMe (LLOMe) in the presence and absence of the peptide aldehyde proteasome inhibitor MG115 [9], [24], [28]. As controls we used the inducers of pyroptotic cell death, anthrax lethal toxin and LPS/nigericin. We found that the proteasome inhibitor MG115 efficiently blocked necrotic cell death mediated by LLOMe (Figure 1A and 1B). MG115 not only blocked cell death but also proteolysis of the inflammatory protein caspase-1 in LLOMe-treated macrophages (Figure 1C). MG115 also blocked cell death by anthrax lethal toxin, as shown previously [29], [31], [32], [34], [35], but failed to block cell death mediated by LPS/nigericin and by the lysosome-destabilizing agent alum (Figure 1A and 1B). As an additional control, we used the cathepsin B inhibitor CA-074-Me, which blocks all forms of programmed necrotic cell death [9], . Consistent with being a pan-necrosis inhibitor, CA-074-Me blocked cell death mediated by the lysosome-destabilizing agents, alum and LLOMe, and by the pyroptosis inducers, LT and LPS/ATP (Figure 1A). Taken together, our findings indicated that aldehyde proteasome inhibitor MG115 blocks necrotic cell death in an inducer-specific fashion.

### Peptide-aldehyde proteasome inhibitors block LMN while non-aldehyde proteasome inhibitors do not

**Figure 2:**
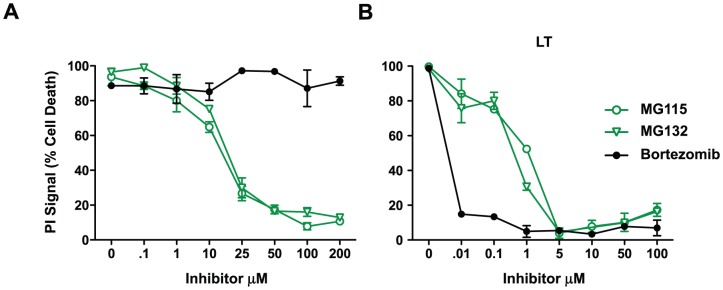
Effect of proteasome inhibitors on cell death mediated by LLOMe and anthrax lethal toxin (LT). BALB/c-derived macrophages were exposed to 2.5 mM LLOMe (A) or anthrax lethal toxin (500 ng/ml PA and 250 ng/ml LF) (B) in the presence of increasing concentrations of the aldehyde proteasome inhibitors, MG115 and MG132, and the non-aldehyde proteasome inhibitor bortezomib. Cell death was measured by PI exclusion two hours after LLOMe/LT exposure. The above data is a representative experiment performed in triplicate.

In order to delineate the role of proteasome in necrotic cell death, we exposed BALB/c-derived macrophages to LLOMe and LT in the presence of increasing amounts of peptide aldehyde and high-potency non-peptide aldehyde proteasome inhibitors. We found that the peptide aldehyde proteasome inhibitors, MG115 and MG132, blocked LLOMe-mediated cell death at concentrations above 25 µM, while inhibitor concentrations as low as 1 µM were sufficient to block LT-induced cell death (Figure 2 and Figure S1). Intriguingly, the non-peptide aldehyde proteasome inhibitors, bortezomib (Velcade) (Figure 2A) and Salinosporamide (data not shown) failed to block LLOMe-mediated necrosis even at concentrations above 100 µM. Consistent with proteasome involvement in LT toxicity, bortezomib blocked LT-induced cell death at nanomolar concentrations (Figure 2B). The failure of bortezomib and Salinosporamide to block LLOMe-induced cell death suggested that cellular systems other than the proteasome control LLOMe-induced necrotic cell death.

### MG115 inhibits cathepsin C activity

**Figure 3:**
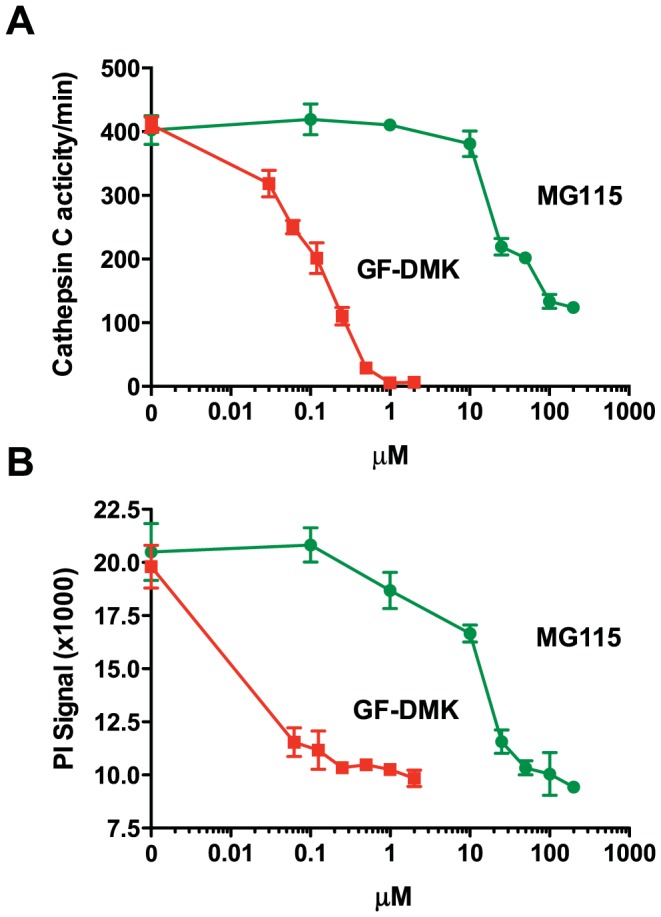
MG115 inhibits cathepsin C activity. (A) The cathepsin C assay was performed in the presence of 1 ng recombinant cathepsin C, the cathepsin C substrate Gly-Arg-AMC, and increasing concentrations of MG115 or the cathepsin C inhibitor Gly-Phe-DMK (GF-DMK). Cathepsin C activity was measured by analyzing Gly-Arg-AMC cleavage at 460 nm. (B) C57BL/6-derived macrophages were exposed to 2.5 mM LLOMe in the presence of increasing concentrations of MG115 or GF-DMK, and cell death was measured by PI exclusion two hours after LLOMe exposure.

Using a haploid screen, we have previously shown that a single protease, cathepsin C, is critical for the induction of cell death and the adaptive immune response mediated by LLOMe [9]. To date cathepsin C is the only critical mediator of LLOMe-mediated cell death that has been identified [9]. As peptide aldehyde proteasome inhibitors block LLOMe-induced cell death in a proteasome-independent fashion, we investigated whether the inhibitors might prevent LLOMe toxicity by directly targeting cathepsin C. To this hypothesis, we incubated recombinant murine cathepsin C with the cathepsin C substrate Gly-Arg-AMC (GR-AMC) in the presence of increasing concentrations of MG115. As postulated, the proteasome inhibitor MG115 blocked the activity of recombinant cathepsin C (Figure 3A), as indicated by reduced release of the fluorescent AMC moiety in the presence of MG115 [37]. We found that MG115 concentrations that blocked cathepsin C activity also prevented LLOMe-mediated cell death (Figure 3A and B). As MG115 blocked cell death and cathepsin C activity at similar concentrations, it was reasonable to assume that the inhibitor blocked LLOMe-mediated cell death by directly targeting cathepsin C. As a positive control, we used the cathepsin C-specific inhibitor, Gly-Phe-DMK (GF-DMK), which efficiently blocked cathepsin C activity at concentrations below 1 µM (Figure 3A). As seen with MG115, GF-DMK blocked LLOMe-induced cell death and cathepsin C activity at similar concentrations (Figure 3A and B). Our studies using MG115 in the recombinant cathepsin C assay were consistent with a central role of cathepsin C in LLOMe toxicity.

### Proteasome inhibitors block lysosome rupture mediated by the lysosome-destabilizing agent LLOMe

As aldehyde proteasome inhibitors blocked recombinant cathepsin C *in vitro*, we next wanted to determine whether these inhibitors also block cathepsin C *in vivo*. Multiple studies have suggested that the carboxypeptidase activity of cathepsin C converts the dipeptide-methyl ester LLOMe into a membranolytic polymer responsible for lysosome rupture and cell death induction [9], [38], [39]. We therefore expected that aldehyde peptide inhibitors block lysosome rupture mediated by LLOMe. To address this question, we exposed primary murine macrophages to the lysosome-destabilizing agents, LLOMe and alum, and to the pyroptosis inducer LT in the presence of proteasome inhibitors. We quantified lysosomal integrity by flow cytometry using the lysosomal pH-indicators LysoTracker and acridine orange (AO). Multiple labs, including our own, have previously shown that the loss of pH gradients in cells treated with alum and LLOMe is concurrent with actual lysosome rupture [9], [24], [28]. We therefore used LysoTracker and AO to monitor lysosome integrity in cells exposed to lysosome-destabilizing agents.

**Figure 4:**
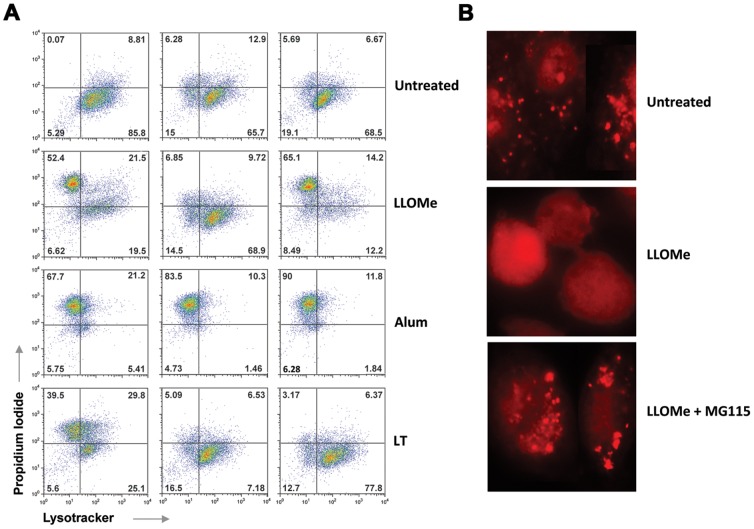
The proteasome inhibitor MG115 blocks LLOMe-mediated lysosome rupture. (A) Flow cytometry analysis of LLOMe-, alum- and LT-treated cells. BALB/c-derived macrophages were exposed to 2.5 mM LLOMe, LT (500 ng/ml PA and 250 ng/ml LF), or alum (150 µg/ml) in the absence and presence of 100 µM MG115 and 50 µM bortezomib. Lysosome and membrane integrity were measured using LysoTracker and PI by flow cytometry 2 hours post LLOMe and LT challenge, and 6 hours post alum challenge. The flow cytometry plots are representative images of two experiments each performed in triplicate. (B) C57BL/6-derived macrophages were exposed to 2.5 mM LLOMe in the absence and presence of 100 µM MG115. Analysis of lysosome integrity was determined by acridine orange (AO) staining 2 hours post LLOMe exposure. The above data is representative of three experiments.

As expected, MG115 blocked lysosome rupture in LLOMe-treated macrophages (Figure 4A). Consistent with earlier findings, this block was specific to the peptide aldehyde proteasome inhibitor MG115, while the non-peptide aldehyde proteasome inhibitor bortezomib failed to block LLOMe-induced lysosome rupture (Figure 4A). Bortezomib, however, prevented lysosome impairment mediated by LT consistent with a role of the proteasome in LT toxicity (Figure 4A). In agreement with previous findings (Figure 1A), MG115 and bortezomib failed to block lysosome impairment mediated by alum (Figure 4A). As an alternative way to measure lysosome integrity, we used the lysosomotropic dye acridine orange (AO). The weakly basic dye acridine orange accumulates within acidic endosomes and lysosomes, and is released upon lysosome rupture. We found that the punctate AO pattern observed in untreated cells progressed to a diffuse cytosolic and nuclear staining upon LLOMe exposure indicating a loss of lysosomal integrity (Figure 4B). As expected, the proteasome inhibitor MG115 prevented the AO diffusion mediated by LLOMe (Figure 4B). Taken together, our findings indicated that peptide aldehyde proteasome inhibitors prevent lysosome rupture mediated by LLOMe-induced cell death, presumably by blocking cathepsin C.

**Figure 5:**
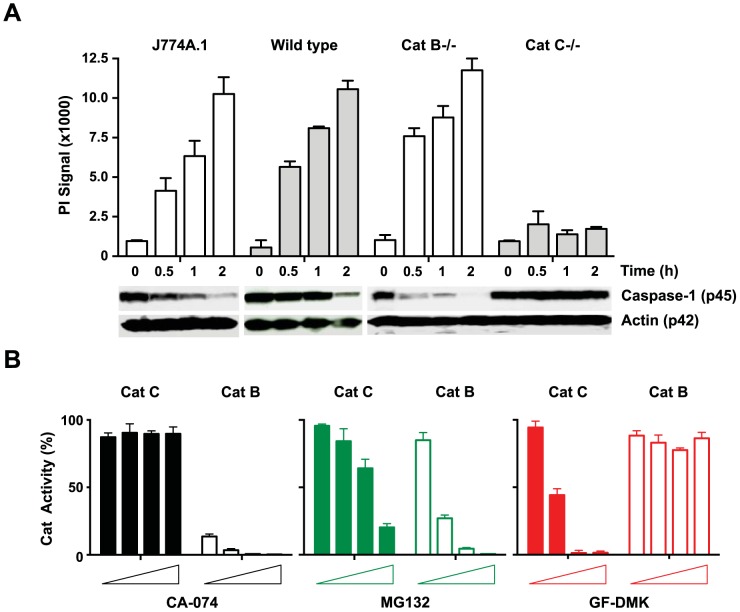
CA-074-Me and Cathepsin C deficiency blocks cell death and protein degradation mediated by LLOMe. (A) J774A.1 macrophages, and wild type, cathepsin B- or C-deficient C57BL/6-derived macrophages were treated with 2.5 mM LLOMe, and cell death was measured by PI exclusion two hours after LLOMe exposure. Corresponding lysates were subjected to immunoblotting and were probed with anti-caspase-1 and actin antibodies (lower panel). (B) The *in vitro* assays were performed in the presence of recombinant cathepsin B or cathepsin C, the corresponding cathepsin B and C substrates, and increasing concentrations (0.01, 0.1, 1 and 10 µM) of MG132, the cathepsin C inhibitor GF-DMK or the cathepsin B inhibitor CA-074-Me. Cathepsin B and C activity was measured by analyzing Gly-Arg-AMC and Arg-Arg-AMC cleavage at 460 nm, respectively.

To determine the contribution of cathepsin C-dependent and -independent events in LLOMe toxicity, we exposed wild type, and cathepsin B-, and C-deficient macrophages to LLOMe. Consistent with earlier studies [9], [24], we found that cathepsin C-deficiency blocks caspase-1 degradation and cell death mediated by LLOMe (Figure 5A). As expected, cathepsin B-deficient macrophages were susceptible to LLOMe-induced cell death and proteolysis (Figure 5A). This is intriguing, as the cathepsin B inhibitor CA-074-Me blocks, like MG115, LLOMe-induced lysosome rupture and necrotic cell death [9], [24].

### CA-074-Me inhibits lysosome-mediated necrosis by inhibiting a factor distinct from cathepsins B and C

The cathepsin B inhibitor, CA-074-Me is the only inhibitor known to block all forms of programmed necrotic cell death [9], [22], [28], [36], including alum and LLOMe-mediated cell death (Figure 1A). Cathepsin B-deficient cells are susceptible to LLOMe-induced cell death (Figure 5A), indicating that cathepsin B is dispensable for LLOMe killing. Accordingly, high concentrations of CA-074-Me, like MG115 and MG132, are required to block LLOMe-mediated cell death [9], [22], [28], [36], suggesting that off-targets are involved in the CA-074-Me block of LLOMe toxicity. We next investigated whether CA-074-Me prevents LLOMe-induced cell death by inhibiting cathepsin C, as observed with MG115. To test this, we incubated recombinant murine cathepsins B or C with their corresponding substrates in the presence of increasing concentrations of CA-074, MG132 and GF-DMK. As CA-074-Me has to be activated by cellular esterase [29], we used its active metabolite CA-074, which does not require esterase cleavage of the methyl-ester group for activation. In contrast to MG115, the cathepsin B inhibitor CA-074 did not block cathepsin C activity even at exceeding concentrations (Figure 5B). CA-074, however, blocked the activity of recombinant cathepsin B at concentrations below 1 µM (Figure 5B), consistent with it being a potent cathepsin B inhibitor. As expected GF-DMK blocked cathepsin C, but not cathepsin B (Figure 5B). Surprisingly, MG132 also blocked cathepsin B, and at concentrations that were even lower than those required for blocking cathepsin C (Figure 5B), indicating that MG132 blocks a wide range of proteases. As cathepsin B-deficient cells are susceptible to LLOMe toxicity, we assume that the MG132 block of cathepsin B is merely an epiphenomenon, and not involved in the MG132 inhibition of LLOMe toxicity.

Here we demonstrated that CA-074-Me blocks LLOMe killing in a cathepsin C-independent fashion, while peptide aldehyde proteasome inhibitors act in a cathepsin C-dependent fashion indicating that both inhibitors prevent LLOMe toxicity by targeting distinct regulators. The knockout experiments also indicated that cathepsin B is also not involved in the CA-074-Me block of LLOMe killing. These findings indicated that the cathepsin B inhibitor targets a novel, yet unidentified critical factor in LLOMe-mediated necrosis.

**Figure 6:**
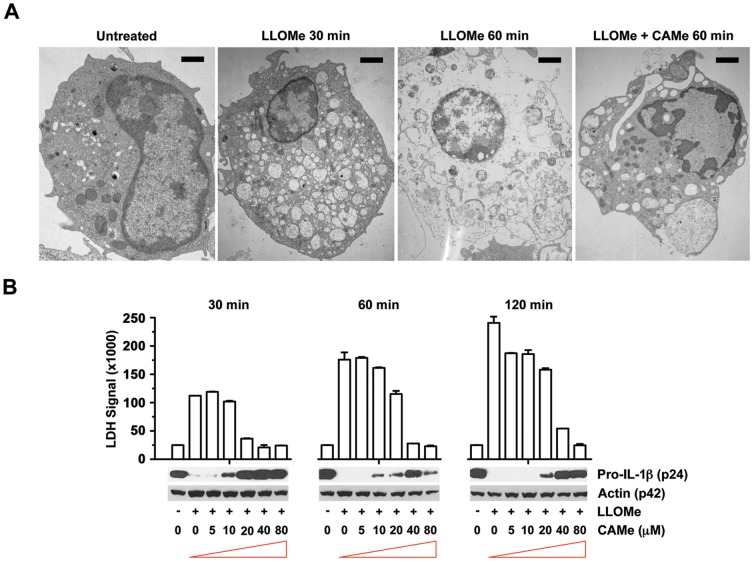
The cathepsin B inhibitor blocks LLOMe-induced necrotic cell death. (A) C57BL/6-derived BMDCs were exposed 2.5 mM LLOMe and analyzed by electron microscopy 30 and 60 min post-LLOMe exposure, respectively. Bars correspond to 1 µm. (B) C57BL/6-derived macrophages were treated with 2.5 mM LLOMe in the presence of increasing amounts of CA-074-Me (in µM). At different time points, cell death was measured by propidium iodide exclusion, and lysates were probed for IL-1β and actin by immunoblotting. All measurements were performed in triplicate.

To further analyze how CA-074-Me prevents LMN, we determined the morphological changes of primary murine macrophages following LLOMe challenge in the presence of CA-074-Me. As observed in necrotic cell death mediated by anthrax lethal toxin [32], [40], LLOMe triggered morphological changes associated with necrotic cell death, such as a loss of the cellular matrix, loss of organelle structure and the formation of multi-vesicular bodies (Figure 6A). We also challenged primary macrophages with LLOMe in the presence of increasing CA-074-Me concentrations. LLOMe triggered a very rapid onset of membrane impairment, another hallmark of necrotic cell death as measured by PI exchange and LDH release (Figure 6B, and Figure S2), and proteolysis of IL-1β (Figure 6B). Both necrotic events were efficiently blocked by CA-074-Me. In fact, we found a perfect correlation between cell death induction and processing of proinflammatory proteins (Figure 6B), suggesting a direct linkage between these processes in LLOMe-treated cells.

### Summary

Lysosome-mediated necrosis (LMN) is a distinct cell death pathway that could trigger a strong adaptive immune response [9], [11], [12], [24]. Identification of factors controlling LMN will advance our understanding of immune regulators. The phenotype of LMN is characterized by simultaneous membrane impairment and broad proteolysis of proteins [9], [11], [12], [24]. Given the role of the proteasome in multiple forms of necrotic and apoptotic cell death [30], [31], [32], [41] the proteasome presented an attractive regulator for the LMN phenotype. Here we describe that peptide proteasome inhibitors efficiently block lysosome rupture, degradation of proinflammatory proteins and necrotic cell death mediated by LLOMe. However, peptide proteasome inhibitors do not act by blocking the proteasome, but rather through pleotropic effects by directly targeting the key regulator of LLOMe toxicity, cathepsin C. Previous studies have demonstrated that cathepsin C converts the dipeptide-methyl ester LLOMe into an amphipathic peptide polymer that then destabilizes lysosomes resulting in LMN [9], [11], [12], [24], [38], [39].

Like aldehyde proteasome inhibitors, the pan-cell death inhibitor, CA-074-Me, also blocks LLOMe-induced lysosome rupture, degradation of proinflammatory proteins and necrotic cell death. While peptide aldehyde proteasome inhibitors and CA-074-Me block an upstream event in LLOMe-treated cells, our findings indicated that they have distinct targets. While peptide aldehyde proteasome inhibitors block LLOMe lysosome rupture and necrotic cell death by blocking cathepsin C, CA-074-Me acts in a cathepsin C-independent fashion and targets a distinct, unidentified mediator of LMN. Our findings suggest that CA-074-Me blocks an event leading to cathepsin C activation in LLOMe-mediated necrosis, and an upstream event in necrotic cell death in general.

Intriguingly, CA-074-Me blocks all known forms of programmed necrotic cell death [9], [22], [28], [36]. In addition to preventing LMN, CA-074-Me also blocks pyroptotic cell death upstream of caspase-1 activation [9], [22], [28], [36], and necroptosis upstream of RIP kinase [22], [23]. As with LMN, the doses of CA-074-Me needed to block necrotic cell death are higher than those are needed to inhibit cathepsin B, as well as many other cysteine cathepsins [11], [24]. Our data suggest that the putative target of CA-074-Me may be a common upstream protease and regulator in multiple necrotic cell death pathways, possibly another cysteine cathepsin. However, our studies using a haploid screen and cathepsin-deficient macrophages identified only a single cathepsin, cathepsin C, as essential for LLOMe toxicity [11], [24]. However, our findings indicate that a second CA-074-Me-susceptible protease, in addition to cathepsin C, controls early event in lysosome-mediated necrosis. It remains to be shown whether CA-074-Me targets the same protein in each form of programmed necrosis, and identification of this protein will provide significant insights into the fundamental processes controlling necrotic cell death.

Taken together, our findings indicated that a cascade of proteolytic events controls lysosome rupture and necrotic cell death mediated by lysosome-destabilizing agents and other inducers of necrotic cell death. Identification of these early events will be crucial in our understanding of checkpoints controlling necrotic cell death, and ultimately the biological processes effected by necrotic cell death.

## Materials and Methods

### Chemicals

MG115, MG132 and nigericin were purchased from Calbiochem (San Diego, CA), and bortezomib (Velcade) was a generous gift from Nereus Pharmaceuticals (San Diego, CA). Bafilomycin A was purchased from LC Laboratories (Woburn, MA). Imject alum purchased from Thermo Scientific (Rockford, IL). NH4Cl was purchased from Fisher Scientific (Pittsburgh, PA). Propidium Iodide was purchased from Sigma (St. Louis, MO). CA-074-Me was purchased from Peptides International (Louisville, KY). CA-074 was a generous gift from Kartik Chandran (Albert Einstein College of Medicine).

### Cell Culture

Wild type C57BL/6 and BALB/c mice were purchased from Jackson Labs (Bar Harbor, MN). The BALB/c-derived macrophage line J774A.1 was purchased from ATCC (Manassas, VA). Bone marrow-derived macrophages were generated from bone marrow derived from femurs and tibias of wild type C57BL/6 and BALB/c mice, and from cathepsin-deficient C57BL/6 mice. We used C56BL/6-derived macrophages for most assays, as macrophages from these strains could be harvested with high efficiency, and these cells are highly susceptible to most inducers of necrotic cell death, including LLOMe, alum and LPS/nigericin. However, C56BL/6-derived macrophages are resistant to necrotic cell death mediated anthrax lethal toxin (LT), which kills macrophages in a strain-dependent fashion. We therefore performed all experiments that included LT with BALB/c macrophages, which are highly susceptible to LT toxicity, but still susceptible to LLOMe, alum and LPS/nigericin-induced cell death. Cathepsin-deficient C57BL/6 mice were generously provided by Drs. Johanna Joyce and Thomas Reinheckel [42], [43], [44]. BMMs were isolated and grown for 7 days in DMEM, containing 10% FCS, 20% L929 preconditioned media, 1% HEPES, 1% MEM nonessential amino acids, and 0.1% cell-culture grade BME. For the preparation of primary dendritic cells, bone marrow cells were conditioned in RPMI media containing 10% FCS, 1% HEPES, 1% MEM nonessential amino acids, 0.1% Gibco BME, in the presence of 20 ng/ml GM-CSF (Leukine; Berlex, Montville, New Jersey).

### Cell death assays

Macrophages were plated in 96-well plates at 1×10^6^ cells/ml, and cell death was determined by measuring membrane impairment by analysis of propidium iodide exclusion, or by measuring LDH activity, at specified time points. Propidium iodide (PI) was added to a final concentration of 30 µM 10 min prior to analysis. PI exclusion assays were performed in phenol-red-free DMEM and analyzed with a Wallac 1420 Multilabel Counter, Victor 3 plate-reader (Perkin-Elmer Life, Mountain View, CA). The LDH activity was measured using the CytotoxOne kit (Promega, Madison, WI) according to the manufacturer's description.

### Flow cytometry, immunoblotting and confocal microcopy

For flow cytometry, bone marrow-derived macrophages were labeled with CD11b-PE, lysosome integrity was measured with LysoTracker Green and acridine orange, and membrane impairment was determined by using PI. Cells were fixed in 1% paraformaldehyde, and analyzed using a BD LSRII flow cytometer. For immunoblotting, macrophage supernatants were collected and spun down at 300 g for 10 min at 4°C. For cell lysate preparations, RIPA buffer (Boston Bioproducts, MA) containing protease inhibitor cocktail (Roche, Basel, Switzerland) was added to cells and incubated at 4°C for 10 min. Cell lysates were collected and spun at 13,000 rpm for 10 min at 4°C. Supernatants and lysates were placed in water bath at 100°C for three min, and run on 12% Tris-HCl gels (Bio-Rad). Gels were then transferred onto PVDF membranes with a semi-dry transfer apparatus (Bio-Rad). Membranes were probed with anti-caspase-1, IL-1β and actin antibodies (R and D Technologies, Kingstown, RI). Antibodies against goat were obtained from Santa Cruz (Santa Cruz, CA). Confocal microcopy using acridine orange was performed as described previously [44].

### *In vitro* cathepsin cleavage assays

Recombinant active murine cathepsin B and cathepsin C were purchased from R&D Systems (Minneapolis, MN), and the *in vitro* cathepsin assays were performed according to the manufacturer's description. The cathepsin B substrate Arg-Arg-AMC and the cathepsin C substrate Gly-Phe-AMC were purchased from Bachem (Torrance, CA), and the cathepsin C inhibitor, Gly-Phe-diazomethylketone (Gly-Phe-DMK), was from MP Biomedicals (Solon, OH). The cathepsin B assay was performed with 5 µg of recombinant active murine cathepsin B in the presence of 200 µM of the cathepsin B substrate Arg-Arg-AMC and various inhibitors. Cathepsin B was activated with 1 mM dithiothreitol and 0.5% Triton X-100 for 1 hour. The cathepsin C assay was performed with 1 µg of recombinant active murine cathepsin C in the presence of 200 µM of the cathepsin C substrate Gly-Phe-AMC and various inhibitors. The generation of free AMC was determined by measuring excitation and emission wavelengths of 380 nm and 460 nm for 10 min at 30°C using a Victor 3 plate-reader (Perkin-Elmer Life, Mountain View, CA). Cathepsin B and C activity was determined by measuring the slope of the increase in AMC fluorescence over time.

### Transmission electron microscopy

Samples were fixed with 2% glutaraldehyde in sodium cacodylate buffer, pH 7.4, and post fixation was performed with 1% osmium tetroxide, followed by 2% uranyl acetate treatment as described previously [44]. After dehydration through a graded series of ethanol washes, samples were embedded in LX112 resin (LADD Research Industries). Ultrathin sections were cut on a Reichert Ultracut UCT, stained with 25% uranyl acetate followed by lead citrate, and examined with a JEOL 1200EX transmission electron microscope at 80 kV.

### Ethics statement

The animal study protocol (#503-2012) was reviewed and approved by the Animal Care and Use Committee of the National Institute of Allergy and Infectious Diseases, National Institutes of Health.

## Supporting Information

Figure S1 BALB/c-derived macrophages were exposed to anthrax lethal toxin (LT: 500 ng/ml PA and 250 ng/ml LF) in the presence of increasing concentrations of MG115 (A), MG132 (B), or bortezomib (C). Control cells were exposed to inhibitor only. Cell death was measured by PI exclusion two hours after LT exposure, and relative cell death levels are shown. The above data was performed in triplicate.
(EPS)

Figure S2 C57BL/6-derived macrophages were exposed to 2.5 mM LLOMe in the presence and absence of 100 µM CA-074-Me, and cell death was measured by LDH release (A) and PI exclusion (B) at different time points post LLOMe exposure.
(EPS)
